# The Impact of the Sex of Handlers and Riders on the Reported Social Confidence, Compliance and Touch Sensitivity of Horses in Their Care

**DOI:** 10.3390/ani11010130

**Published:** 2021-01-08

**Authors:** Ashley Anzulewicz, Kate Fenner, Michelle Hyde, Susan Heald, Bibiana Burattini, Nicole Romness, Jessica McKenzie, Bethany Wilson, Paul McGreevy

**Affiliations:** 1 Sydney School of Veterinary Science, University of Sydney, Camperdown, NSW 2006, Australia; kfen3285@uni.sydney.edu.au (K.F.); michellehyde1@bigpond.com (M.H.); bbur2426@uni.sydney.edu.au (B.B.); nrom3994@uni.sydney.edu.au (N.R.); jmck2527@uni.sydney.edu.au (J.M.); Bethany.wilson@sydney.edu.au (B.W.); paul.mcgreevy@sydney.edu.au (P.M.); 2 Community Health Sciences, University of Manitoba, Winnipeg, MB R3T 2N2, Canada; sheald53@gmail.com

**Keywords:** equine behaviour, rider sex, horse welfare, welfare

## Abstract

**Simple Summary:**

Any influence of the sex of the human partner in human–horse interactions on the behaviour of horses is currently largely anecdotal. Associations between the sex of humans and equine behaviour may have welfare implications. Our study investigates observations of ridden and non-ridden horse behaviour, as reported by respondents (*n* = 1420) to the Equine Behaviour and Research Questionnaire (E-BARQ). Results reveal some human sex-related differences between horses handled and ridden by male and female humans. Horses ridden or handled by male humans were more likely to be difficult to catch and defensive when approached, but less likely to pull on the reins/brace the neck or toss their head. The study revealed the importance of considering the sex of the rider or handler when investigating equine behaviour.

**Abstract:**

Current evidence of how human sex-related differences in riders and handlers may influence horse behaviour is limited. The Equine Behaviour Assessment and Research Questionnaire (E-BARQ) was used to collect demographic data on riders and handlers (*n* = 1420) and behavioural data on their horses. It includes demographic items about the sex of the respondent and how frequently the horse has been ridden or handled by male and female humans. The questionnaire then gathers observations on the horse’s behaviour on the ground and under saddle or when driven. Using E-BARQ’s battery of 97 questions, the current study showed differences in ridden and non-ridden horse behaviour that were related to the sex of the rider or handler. Data were evaluated using multivariate analysis and revealed that horses handled by male humans were significantly more difficult to catch (*t*-value = −3.11; *p* = 0.002) and significantly more defensive when approached (*t*-value = −2.104; *p* = 0.035), but significantly less likely to pull on the reins/brace the neck or toss their head (*t*-value 1.980; *p* = 0.048) than horses handled more frequently by female humans. The differences found between male and female horse handlers suggest that sex is an important factor to consider when understanding equine behaviour. Our study explored reported differences in confidence, handling and working compliance and touch sensitivity among horses ridden and handled by male and female humans and suggested further research into how these differences are gendered.

## 1. Introduction

Since their domestication approximately six thousand years ago [1], horses have played important roles in human society. These roles have changed over time. Horses previously played a critical role in the military, in agriculture and transport [2]. Since the invention of the internal combustion engine, horses have transitioned into more modern roles such as sport, entertainment, leisure and companion animals [1,3]. As horses have continued to play a prominent role in the lives of humans, the need to assess and assure equine welfare has become increasingly important [4]. The ability to understand and react to equine behaviour may influence the welfare of horses with perceived behavioural problems [5]. These horses are often subjected to excessive or prolonged pressure as part of poorly applied negative reinforcement, but may also be subjected to physical punishment that, especially if unsuccessful, often escalates into violence [5]. These techniques may cause fear and avoidance, which make horses increasingly difficult and ultimately may render them dangerous, not least when horses buck, bolt and rear [6]. Due to their unwelcome behaviours and safety concern, such animals often change hands, with minimal exchange of their case histories that brief new owners on how they have been handled and by whom [5]. In recent years, considerable research attention has turned to the role of the human partner in creating successful human–horse relationships [7]. However, the impact that rider or handler sex might have on equine behavioural traits is largely unknown.

In general terms, and for the purposes of this article, “sex” refers to the biological differences between males and females. Sex is usually assigned at birth by the appearance of the external genitalia or sometimes by genetic differences, should these be known. “Gender” refers to the ways that masculinity and femininity are performed in negotiation with group norms. Gender is context-specific. For example, in the context of self-advocacy, women have been found to curtail their assertiveness and thus obtain lower outcomes, whereas in other advocacy contexts, they achieve better outcomes resulting from less inhibition [8]. Influencing contexts may range from broad social categories such as nation, race and sexual orientation, to familial and partner situations, further, to subgroupings such as equestrian sport. These subgroups in turn can be broken down by discipline, such as by whether people participate in competitions and at what level, but also by smaller subgroups, such as a particular riding arena or boarding stable. Individuals, whether male, female or intersex, can conform more or less to dominant models of femininity and masculinity, by choice, by upbringing and by phylogenetic traits [9,10,11].

To understand how the sex of the rider or handler may influence equine behaviour, it is important to understand the physiological differences between male and female humans [12]. Such differences between male and female humans stem from variations in circulating concentrations of sex hormones. Sex steroid receptors are found in numerous non-reproductive tissues, including the brain [13]. Their presence influences how male and female humans differentiate between factors such as pain and stress [13,14]. Male and female humans have also been reported to differ in range of motion and gait, with female humans walking with a shorter stride length and slower speed than male humans [15]. These differences extend into further musculoskeletal differences, including range of motion in joints, such as the hip and ankles [16]. Female humans have less range of motion in their hips but greater range of motion in their ankles when compared to male humans [17]. Whether these differences relate to the way in which male and female humans interact with animals is still not completely understood. Correlations have been made between the behaviour of people handling animals and the resulting behaviour of animals, such as in the example of lower milk yield being associated with animal handlers who acted more negatively towards dairy cows, when compared to animal handlers who acted with more neutral behaviour [18]. Even with touching animals, male and female humans can produce different outcomes. For example, in working sheep dog trials, female handlers (*n* = 22) worked their dogs through certain elements of the course quicker than male handlers (*n* = 38) [19].

If differences in behaviour between horses handled by male and female humans exist, they may have welfare implications, because equine welfare is influenced by horse–rider interactions [20]. The resultant information could also help to inform training and management decisions for horse owners and caregivers. These decisions may include re-homing considerations and modified training methods, both of which could facilitate the formation of successful horse–human relationships.

A horse’s confidence is an important factor in determining how competitive the horse is and how well it performs [21]. Training and management appear to have a direct impact on how confident a given horse might be—for example, when prompted to jump a fence or when interacting with livestock or traveling alongside motor vehicles—because confidence is determined by all of the animal’s past experiences [21]. Horses that are routinely forced to work beyond their physical or mental ability often become stressed, frustrated and possibly even injured. As a result, they will begin to seek ways to avoid being trained and handled, which can lead to dangerous behaviours, risking both horse and rider safety [21]. Coercing a horse beyond its physical or mental capabilities, or working a horse in pain, will most likely require the use of aversive stimuli and punishment, such as whipping [22]. Punishment can lead to fearful, defensive or even aggressive behaviours in the horse, ultimately putting the horse and handler at risk [22]. There is some evidence that male observers are more supportive of whip use in racing than female observers [23].

Human touch, and its putative impact on horse behaviour, has been studied in several contexts [24,25,26,27]. It has been shown that optimal touch during handling decreases nervous tendencies in young horses [26]. Along with the act of touching, the way in which a horse is approached also has a direct impact on its behaviour [28]. A recent study investigated the handler’s approach together with the horses’ sensitivity to touch and suggested that horses prefer a human approaching with a submissive posture rather than a more dominant stance [28]. Additionally, horses can use facial expressions and voice to interpret the emotional state of the human approaching them [28]. These findings may suggest that a better level of familiarity among horses and humans could play a beneficial role in allowing horses to better interpret and read human postural and verbal cues and display more appropriate responses in return.

The ability of horses to react to human cues reflects their visual acuity [29] and high level of responsiveness [28]. The compliance of horses in the human domain refers to the extent to which horses respond to anthropogenic stimuli appropriately and consistently [24]. Compliance is most fully tested when a horse is in a potentially stressful or unusual situation, such as when being ridden in a competition or on an unfamiliar trail [24]. A horse’s level of compliance may indicate whether that horse is predictable, or even safe, for a rider/handler to work with. It has been suggested that the level of compliance seen among horses is largely dependent on the horse–rider relationship [24]. The training methods and equipment used on a horse also play a role in determining how compliant a given horse may be [30]. The feminist ethic-of-care tradition would also suggest that the sex of the handler or rider influences the behaviour of the horse. A recent study revealed that male humans were 2.88 times more likely to use spurs while riding, compared to female humans [31]. Spurs may enhance compliance by decreasing a horse’s reaction time to a physical cue to go faster or change directions [31]. However, when spurs are used excessively or not appropriately, habituation may occur, and an escalation of force may ensue. Under such circumstances, equine welfare is negatively affected, as horses may suffer from flank sores and abrasions [31].

Most domesticated horses in developed countries are used for recreational riding, which has not been the primary population group for most historic equine research [32]. This gap in knowledge has highlighted the need for a systematic method for riders and handlers to evaluate equine behaviour. The Equine Behaviour Assessment and Research Questionnaire (E-BARQ) is an ongoing project at the University of Sydney that allows horse owners to benchmark their horse’s behaviour against other horses in the database and to observe changes in their horse’s behaviour over time, in order to track progress [33]. The questionnaire acts as a tool for monitoring the efficacy of various training and management techniques, which may play an important role in improving understanding of equine behaviour and in enhancing equine welfare [34].

Investigations into whether the sex of humans has an impact on equine behaviours associated with the horses’ confidence, handling compliance and touch sensitivity may reveal that the sex of humans contributes, at least in part, to the origins of equine behaviour. Understanding the behavioural tendencies of any given horse may allow potential owners to understand the horse’s specific needs and better tend to these. The aim of the current study was to explore the differences in confidence, handling compliance and touch sensitivity of horses ridden and handled by male humans and female humans, as reported through the E-BARQ.

## 2. Materials and Methods

The project was approved by the University of Sydney Human Research Ethics Committee (approval number: 2012/656).

E-BARQ, designed to draw objective data, is a not-for-profit, validated [34], equine behavioural questionnaire created on the Qualtrics platform [35]. The questionnaire consists of 97 matrix-style questions, which include 42 demographic items about the horse owner and handler. The questionnaire then divides into 268 items for the ridden or driven horse and 218 items for the non-ridden horse [33] (see Supplementary File S1). E-BARQ is an ongoing project and, for the purposes of the current study, was distributed to horse owners, riders and trainers through social media platforms such as Facebook and Instagram. It was distributed via social media, equestrian sport organisations’ email contacts and the email lists of *Horses and People Magazine* (https://horsesandpeople.com.au/), *Equitation Science International* (https://www.esi-education.com/) and *Kandoo Equine* (https://www.kandooequine.com/). To optimise male respondent recruitment, equestrian sport organisations with male riders, such as working cow horse, reining and show-jumping, were specifically targeted.

### 2.1. Trait Selection

During the development of E-BARQ, a rotated principal component analysis of 218 behavioural, management and training questions extracted a total of 65 rotated components [33]. Twenty-four E-BARQ items (that combined ridden and unridden horse questions) featured in the eight underlying rotated components of interest. To combine these into fewer relatively uncorrelated indices, a parallel analysis, comparing the rotated components of the standardised observed data with those of a random data matrix of the same size, was used and obtained through Psych function of R statistical software (Revelle, W. (2019) psych: Procedures for Personality and Psychological Research, Northwestern University, Evanston, IL, USA, https://CRAN.R-project.org/package=psych version 1.9.12.). Kaiser, Meyer, Olkin Measures of Sampling Adequacy (MSA) and Cronbach’s alpha (α) were also considered using the Psych package.

Indices were constructed by assigning a numerical value to scores on the five-point Likert scale of the relevant E-BARQ items (see Supplementary File S1). Once assigned, these values were summed together. In the case of missing values, the sum was divided by the number of E-BARQ items in the index, for which the horse’s information was available, and multiplied by the number of items used to calculate the index. This weighted the missing value according to the horse’s score for similar items rather than imposing an overall mean. If no E-BARQ items for a given index were completed, then a value for that horse was not calculated.

The six underlying rotated components, and their associated questions, analysed in order to evaluate the effect of gender on equine behaviour, are summarised in Table 1.

Other predictor variables available (including potential confounders) were assessed for potential inclusion in the final model by univariate analysis, along with the above indexes. Univariate logistic regression models were applied to assess whether the E-BARQ indices and demographic variables would predict gender. Demographic variables included country of rider, age of rider, laterality of rider, sex of horse, age of horse, colour, height of horse, breed, rider experience, discipline, human social confidence, intervention compliance, head compliance, bridling compliance, catch compliance and absence of defensive aggression. Non-index predictors, apart from breed, with a *p*-value of <0.3 on univariate analysis were passed into the multivariate model building process. Because of strong multicollinearity with discipline [1], breed was discarded. Index predictors with a *p*-value of <0.7 were passed into the multivariate model building process.

### 2.2. Multivariate Modeling

In the respondent human sex model, the dependent variable was sex of survey respondent, and the six potential explanatory variables were human social confidence index; intervention compliance index; head compliance index; bridling compliance index; catch compliance index; and absence of defensive/aggressive index. Country of rider, age of rider, laterality of rider, sex of horse, colour, height of horse, rider experience and discipline were also included [6].

A second model, to assess whether the indices and demographic variables selected by univariate analysis, would predict variation in the frequency of handling by male humans. This was developed using the MAAS statistical analysis package [36], and residuals were assessed graphically. The frequency of handling by male humans was modelled and the frequency of handling by female humans was also added to control for the frequency of handling itself. The parallel log odds assumption was assessed graphically and with a likelihood ratio test from the ordinal package [37]. Surrogate residuals were generated by the Sure package [38] and assessed graphically.

## 3. Results

Responses from 1361 female participants and 59 male participants were explored for the purpose of this study. Horse owners and caregivers from 33 different countries completed the questionnaire. Sixty percent of reported horses were purebred animals, representing more than 78 different breeds. Data represented 38% mares, 58% geldings and the remainder stallions, colts and fillies. More than 83% of respondents had over eight years of riding or horse handling experience. Ninety percent of respondents were aged between 18 and 64 years of age, with more than 80% of those reporting having owned or worked with horses before the age of 16 years.

During the E-BARQ survey, participants were able to state whether their horse was ridden/handled by the opposite sex, and if so, how frequently in the preceding this occurred. The results for the question about how often horses were handled by men/boys and women/girls are outlined below in Table 2.

The components were separated into subsections classified as temperament (T) and equitation (E). Results of the parallel analysis are presented in Table 3. These temperament and equitation variables of interest were underlying components identified during the development of E-BARQ [33]. Given the subsequent modification of E-BARQ that took place in light of development feedback, and to ensure that these components were still valid, the underlying questions comprising these components were subjected to a parallel analysis. This determined the number of components to extract and was followed by a principal component analysis to allow comparison to the constructs extracted from the original E-BARQ.

Indices were constructed by assigning a numerical value to scores on the Likert scale (see Parameterisation above) for the relevant E-BARQ items and summing these values together. In the case of missing values, the sum was divided by the number of E-BARQ items in the index for which information for that horse was available and multiplied by the number of items used to calculate the index, weighting the missing value according to the horse’s score for similar items rather than imputing an overall mean. If no E-BARQ items for an index were completed, then a value for that horse was not calculated. The six indices were grouped as follows: T24—Human Social Confidence index; T3—Intervention Confidence index; E1—Head Compliance index; E7—Bridling Compliance index; E9—Catch Compliance index; T5, T15, T17 and T21—Defensive-Aggressive index.

The overall MSA value for T24 was 0.66 and Cronbach’s alpha was 0.75 (95% confidence interval (CI) = 0.73–0.77), the latter of which was not improved by dropping any item. The overall MSA value for T3 was 0.75 and Cronbach’s alpha (α) for T3 was 0.83 (95% CI = 0.82–0.84), the latter of which was not improved by dropping any item. For E1, the overall MSA value was 0.78 and Cronbach’s alpha (α) 0.77 (95% CI = 0.68–0.73). Removing E7 (pull back when unbridled) slightly lifted the alpha to 0.71. Therefore, given the small improvement relative to the CI, and the small number of items in the index, the item was retained. The overall MSA value for E9 was 0.50 and Cronbach’s alpha (α) was 0.54 (95% CI = 0.5–0.58), the latter of which was not improved by dropping any item. Lastly, the defensive/aggressive components (T5, T15, T17, T21) had an overall MSA value of 0.71 and Cronbach’s alpha (α) was 0.72 (95% CI = 0.70–0.74), the latter of which was not improved by dropping any item.

Other predictor variables, including potential confounders, were assessed for potential inclusion by univariate analysis. Non-index predictors with *p*-value < 0.3 on univariate analysis were passed into the multivariate model building process and are outlined below in Table 4, while the significant traits appear in Table 5.

The final model, a logistic regression [26], was applied to assess whether the indices and demographic variables selected by univariate analysis would predict variation in sex of the rider, expressed as a binary variable. A stepwise procedure was applied by removing the least significant variable until doing so resulted in an increase in the Akaike Information Criterion (AIC). The results showed that catching compliance, human social confidence and head compliance were significantly predicted by human sex.

**Figure 1:**
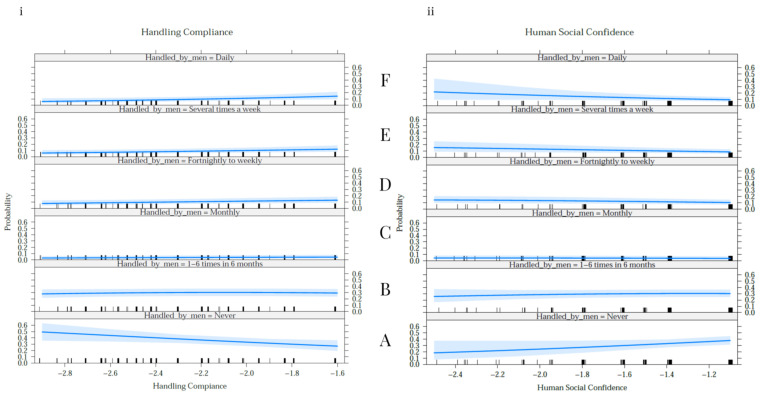
The probability of handling compliance and human social confidence among horses handled by male humans. Each plot shows the frequency with which the reported horse was handled by male humans (A: never, B: 1–6 times in 6 months, C: monthly, D: fortnightly to weekly, E: several times a week, F: daily). The *y*-axis is the probability of handling by male humans, and the *x*-axis (**i**) the size of variance in handling compliance and (**ii**) human social confidence. As the frequency of handling by male humans increases, handling compliance increases and human social confidence levels in the horse decrease.

For the non-ridden horse questionnaire items, respondents classified how frequently their horse was handled by male humans in terms of “never”, “1–6 times in 6 months”, “mostly”, “fortnightly to weekly”, “several times a week” or “daily”. The probability of horses reported as being socially confident towards humans is presented in Figure 1ii, confirming that as the frequency of handling by male humans increases, the social confidence of the horse decreases.

For the non-ridden horse questionnaire items, respondents classified how frequently their horse was handled by male humans in terms of “never”, “1–6 times in 6 months”, “mostly”, “fortnightly to weekly”, “several times a week” or “daily”. The probability of horses reported as being compliant is presented in Figure 1i, showing that, as handling by male humans increases, handling compliance increases.

## 4. Discussion

The purpose of the current study was to investigate possible differences in the behaviour of horses ridden and handled by male and female humans, as reported through the E-BARQ. The results reveal significant relationships between the sex of humans and three equine behavioural traits. Horses ridden or handled by male humans are more likely to be reported to be difficult to catch and more defensive when approached, but less likely to pull on the reins/brace the neck or toss their head than those handled by female humans. Our results show that the probability of human social confidence decreases as handling by male humans increases. Conversely, the probability of handling compliance was seen to increase when handling by male humans also increased.

The current study revealed that horses ridden or handled by male humans are more likely to be reported to be difficult to catch than those ridden or handled by female humans. As female humans have been reported to locomote with a smoother or more rhythmic gait than male humans [17], it is possible that variations in gait may affect the way in which male and female humans approach horses. Studies have suggested that the appropriate manner to approach a horse is at a 45-degree angle and with a slow gait [25]. The longer and less smooth gait reported in male humans may help to explain why horses ridden or handled by male humans are more likely to be reported as difficult to catch.

Defensive behaviours observed among horses were reported by male or female human E- BARQ respondents through three human social confidence questions. The questions captured the degree of agonistic behaviour that their horses displayed when approached in the paddock, in the stable or while eating. The current data revealed that horses handled by male humans were more likely to be defensive when approached. It can be hypothesised that the confidence of these horses may have been compromised at some point in their lives [22] because they lack the traits that E-BARQ analyses cluster together under the label “human social confidence”. Alternatively, it could be posited that the reported defensive behaviours are responses to the more assertive behavioural postures adopted by male humans [39]. It has been suggested that horses prefer to be approached by humans adopting a submissive posture, which is characterised by a lack of eye contact and relatively slow movement [28]. As a prey species, horses may have associated more dominant human locomotory attributes with the stalking behaviour typical of a predator, and so, they may default to the fight-or-flight response typical of unrestrained equids when exposed to potential threats [28]. The defensive behaviours reported more frequently towards male humans may reflect increased vigilance or their innate fight-or-flight response. Although the innate fight-or-flight response is an important consideration in understanding equine behaviour, it is necessary to understand that the behavioural responses displayed by horses vary, along with the salience of what we do to them [40]. It is understood that sufficient familiarity allows horses to respond appropriately to intentional and unintentional human behavioural cues [28]. The same may be said when determining how a horse may react to an asserted or submissive person catching them in a stall or paddock. If a horse is caught by an assertive person every day, it may habituate to any perceived aversiveness and thus become less likely to greet the handler with a flight response, when compared to a horse who is caught by an assertive person only occasionally [40].

In horse training parlance, compliance is a term used to describe how well trainees respond to cues from the humans handling, training and riding them. It can be evaluated by how horses respond when placed in challenging circumstances, such as being ridden in a competition [24]. For the current study, compliance was evaluated in the context of the horses’ responses to various cues both in-hand and under saddle. The current results revealed that horses handled by male humans were significantly less likely than those handled by female humans to pull on the reins/brace the neck or toss their head, which is indicative of more compliance. It is possible that male humans, often being physically stronger than their female counterparts, exert greater tension with rein use, which could result in less response-trialling, such as head tossing, from the horse. Differences in rein tension exerted by male and female riders would be an interesting area for further research.

It is important to consider how some training and handling methods may inadvertently condition a horse to be relatively less compliant or more likely to toss its head. For example, spurs may be used to improve responsiveness to physical cues for a horse to go faster or change direction quickly in a bid to enhance compliance under saddle and competitive performance [31]. It is possible to speculate that compliance under saddle could inherently contribute to the welfare of the horse being ridden, if the rider removes the pressure as soon as the horse responds. Negative reinforcement of desirable responses avoids habituation, which may prompt an escalation of pressure. In the case of bit pressure, this helps in the avoidance of a so-called hard mouth [41] while, in the case of excessive spur use, it avoids the risk in the horse becoming “dead-to-the leg” (also labelled sluggish) and the prospect of flank abrasions [31].

It is important to acknowledge that E-BARQ is anonymous and conducted online, and as such, respondents are left to answer truthfully and to their best ability. Therefore, it is possible that some degree of respondent bias may be reflected in the current results. Specifically, some owners may have been tempted to report the behaviour of their horse more positively (or indeed negatively) than an outsider might. Moreover, the current use of equitation science mailing lists may have led to a form of selection bias, as these targeted individuals who are interested in evidence-based ethical equitation [42]. The authors acknowledge the need for caution when interpreting data from a relatively small number of male respondents. The effects of weather on observations are likely to be cushioned by the E-BARQ’s request for respondents to reflect on the preceding six months of observations and its recording of which hemisphere data are originating from. The current data are likely to reflect the observations of amateurs more than professionals. The relative distribution of male and female riders across equestrian disciplines may merit consideration when the interaction between sex and equestrian discipline is being explored in future studies.

The relatively large number of female respondents in the current study is likely reflective of the true distribution of riders and handlers in the equestrian population, as longitudinal trends within the industry population reflect a dominance of females in recreational riding/handling [43]. Further, because female respondents (*n* = 1361) outnumbered male respondents (*n* = 59), the study may have suffered from low numbers of male respondents. However, all respondents also provided data on the frequency with which males and females handled or rode the focal horse.

In the current study, human sex was explored within a binary system of male or female. The E-BARQ contained one question that allowed respondents to select male, female, gender non-conforming or I’d rather not say. The authors acknowledge that this question confuses “sex” with “gender” and fails to attend to the portion of the human population who are neither male nor female [44]. This question has since been updated in the E-BARQ survey to offer respondents the following choices: male, female, neither or I’d rather not say. For the current study, E-BARQ respondents were also asked how frequently a given horse was handled or ridden by men/boys and by women/girls. Although respondents may not have been aware of it, men, boys, women and girls are categories of gender, not sex.

In interpreting the findings of the current study, it is important to consider the impact of riders’/handlers’ gender, and not sex alone. The concept of gender incorporates societal and subgroup rules and expectations about how people should perform their biological sex, as well as individuals’ efforts to conform to or challenge those expectations [45]. For example, the anatomical differences noted in the introduction of this article, which affect how male and female humans walk [16], are refined over a lifetime by pressures on, and efforts by, boys and men to not walk like a girl. Similarly, consider the pressures on, and efforts by, girls and women to sit in ways that are considered to demonstrate modesty, a feminine trait [46]. The ways that people move, in addition to the ways in which they use their voice, choose their clothing and relate to others, are further examples of human characteristics affected by social forces that are laid over physical bodies. While, in most social contexts, gender is assumed to align with sex, where male bodies express traditional heteronormative masculine traits and female bodies express traditional heteronormative feminine traits, the gender expression of actual male and female humans may not conform to these dominant, binary models of gender. This may be due to subcultural norms or individual choice. Examples in the equestrian world might include physical strength and body size. For example, women in heteronormative societies are often assumed and expected to be physically weaker than men. However, horse handling and equestrian sports are often very physically demanding. Similarly, while large stature and body bulk may be considered as attractive masculine gender traits for men in heteronormative societies, these are not considered assets for male jockeys [27].

Some differences in gait are due to genetic kinematic differences [16]; others are likely learned behaviours, with a cultural influence [15]. As has already been suggested, these gendered ways of approaching a horse may trigger a response in horses such that more traditional heteronormative masculine forms of approach are more likely to result in evasion by horses. Similarly, pulling on the reins, bracing the neck or tossing the head are considered unwanted and even, by some, albeit erroneously, disrespectful behaviours [47,48]. The expectation to be treated with respect is itself gendered, as has been found in research investigating the perceptions of women who confront sexist remarks [49]. As a result, we can expect to encounter differences in handlers’ and riders’ reactions to this so-called disrespect; here, the human’s reaction is to demand respect with harsh or punitive actions—again, perhaps a traditionally considered characteristic of masculinity more than femininity [50]—horses may show passive behaviours or drift into learned helplessness [51], resulting in less observed (and reported) head-tossing and rein-pulling. Equally, head-tossing and reefing at the reins can be learned by horses that seek comfort or autonomy, so it would pay to explore the extent to which they may be manifestations of any differences in the consistency with which males and females use reins and lead-ropes. Certainly, there is merit in assessing how masculine and feminine handlers use leads [52]. Gendered differences may also result in differences in reporting. For example, a handler who expects obedience (masculine) is more likely to consider a horse hard-to-catch than one who expects to need to cajole and entice (feminine) [45,50,53]. The sex of the horse may also influence its behaviour [14] and the relationship between the sex of the horse and that of the handler requires further investigation.

The current findings cannot be explained by sex alone—the concept of gender is needed. In addition, we need more information about the gender expression of both the current E-BARQ respondents and humans who ride and handle horses more generally. Another line of enquiry suggested by the concept of gender relates to the gendering of horses by humans. In 1974, Rubin et al. demonstrated that parents of newborn human babies refer to male babies as big and strong and female babies as small and delicate, even when all objective measures of size and strength are identical [54]. Thus, a fruitful line of enquiry might be to explore the extent to which riders and handlers judge a mare to demonstrate feminine characteristics, while geldings or stallions are seen to be masculine. This would then allow us to explore how expectations that a horse’s behaviour is gendered may result in that horse being treated and trained differently and, in turn, leading to different results on the E-BARQ.

Research in the feminist ethic-of-care tradition—for example, Donovan and Adams [55]—adds another layer to our understanding of how the subjects of the current study are gendered. Grounded in Carol Gilligan’s landmark *In a Different Voice* [56], and in a rejection of hierarchies of domination (male/female; human/animal, mind/body, reason/emotion, nature/culture) explored by Haraway [57,58] and Adams and Gruen [59], this tradition asks political questions about the context in which animals are (mis)treated [55], about how the world would look from animals’ perspectives [60,61], and considers not just the rights of animals, but humans’ responsibilities to, and relationships with, them [62,63]. Gilligan [56] argues that women’s morality would be based on a tradition of care for others. As we have argued here, the fluidity of gender means that both male bodies and female bodies can approach horses with an ethic-of-care. Doing so takes the horses’ perspective into account and challenges handling practices that would subsume animals to the wishes of humans. We have shown here both that handling and riding influence horse welfare and that handling and riding are gendered. This points to the need to further investigate how and with what results. As we look for new ways to understand the relationships among horses, male humans and female humans, the feminist ethic-of-care tradition points to the need to understand how these relationships are themselves gendered and how gender has played a part in developing horse handling practices and traditions.

Gender, as a concept, thus allows us to consider that both male and female humans can and do express themselves in ways that are considered more or less masculine or feminine. What the current study is unable to disclose is how human gender mediates any horse’s behavioural response. As gender is both fluid and constructed, once we know more about how the human behaviours that affect horse behaviour are gendered, riders and handlers can learn to attend to and change their performance of gender in order to bring about better training and welfare outcomes.

Our results show that the probability of horses showing social confidence around humans decreases as handling by male humans increases. Conversely, the probability of handling compliance increases with increased handling by male humans. Handling compliance, as measured by standing for maintenance procedures, head handling, bridling and catching, describes a set of desirable behavioural traits that may make the horse more manageable, particularly on the ground. However, an increase in handling by male humans also resulted in a lower probability of human social confidence. Human social confidence, as measured by defensive or aggressive behaviours displayed by the horse when approached in the field, stable or when eating, can be an important predictor of handler safety. Further research will be required to investigate this relationship and discover ways to balance confidence and compliance in horses and thus optimise horse welfare and handler safety.

## 5. Conclusions

The study revealed apparent differences in horse behaviour as reported by male and female E-BARQ respondents. Horses ridden or handled by male humans are significantly more likely to be reported to be difficult to catch and defensive when approached, but less likely to pull on the reins/brace the neck or toss their head. The current results show that when horses were handled more by male humans, handling compliance increased, but the human social confidence of horses decreased. The results suggest that domestic equine behaviour is influenced by the sex of the rider or handler. However, more study is required to establish whether this influence results from the sex or the gender of the humans. Equine welfare and rider safety can be improved by taking the sex and gender of humans into consideration when seeking to evaluate the origins of equine behaviour.

## Figures and Tables

**Table 1:** The question text used and the question items for each of the six underlying rotated components. The components were separated into subsections classified as temperament (T) and equitation (E).

Question Text	Question Items
Some horses display defensive or aggressive behaviour in certain situations. Typical signs would include threatening to bite, pinning ears, tail swishing, threatening to kick or strike. The most serious signs would include actual biting, kicking or striking. Check a box on the 5-point scale to indicate your horse’s recent tendency (using the previous 6 months as a guide) to show these behaviours in the following context (T24)	Approached by you in the paddock
	Approached by you in the stable
	Approached by you when eating from a bucket or manger
Will [horse_id] stand for (without restraint or when restrained by a head collar and lead rope) (T3)	A general exam by a veterinarian
	Their teeth to be examined by a dentist/veterinarian
	Their feet to be cleaned
	Their feet to be trimmed
	Their feet to be shod
Does [horse_id] (E1)	Raise their head to avoid rein or lead rope cues
	Toss their head when being ridden/driven
	Pull on the reins or lead rope when signals are applied
	Brace their neck when rein or lead rope signals are applied
	Move faster or raise their head when anticipating the transition to canter
Does [horse_id] (E7)	Throw their head up when bridled
	Pull back when bridled
	Pull back when unbridled
Does [horse_id] (E9)	Come when called in the field
	Move away when being caught
Using the last 6 months as a guide, indicate how likely [horse_id] is to display defensive or aggressive behaviour when (T 5, 15, 17, 21)	Hosed down
	The girth is done up
	Verbally corrected when ridden/driven
	Verbally corrected by you or another person on the ground
	Being lunged or worked in a round pen
	Signaled to canter on the lunge

**Table 2:** Outline of responses from The Equine Behaviour Assessment and Research Questionnaire (E-BARQ) question: “In the last 6 months, how often has your horse been ridden or handled by male or female humans?”.

Amount Handled by Female Humans	Handled by Female Humans	Handled by Male Humans
Daily	221	57
Several times a week	628	88
Weekly	212	82
Once a fortnight	77	32
1–2 times a month	118	221
Once a month	45	44
3–6 times in the past 6 months	133	131
Never	104	1167

**Table 3:** The results of the validation parallel analysis suggested the extraction of six underlying rotated components. These were then extracted by a validation principal component analysis using the Psych package of R statistical software and rotated using a varimax rotation, to determine how the E-BARQ indices performed on the new dataset. Items that loaded strongly (>0.60) appear in bold.

E-BARQ Item	RC1 Training	RC2 Husbandry	RC3 Approach	RC4 Catch	RC5 Bridling	RC6 Riding
(T24) Approached in paddock	0.16	0.08	**0.77**	0.2	0.1	−0.04
(T24) Approached in stable	0.12	0.09	**0.84**	0.01	0.06	−0.01
(T24) Approached when eating	0.24	0.01	**0.71**	−0.02	0.07	0.06
(T3) Stand for vet	0.07	**0.74**	0.07	−0.02	0.18	0.1
(T3) Stand for dentist	0.08	**0.66**	−0.03	−0.03	0.11	0.06
(T3) Stand for feet picked	0.09	**0.79**	0.11	0.07	0.11	0
(T3) Stand for feet trimmed	0.08	**0.85**	0.06	0.05	0.05	0
(T3) Stand for shod	0.02	**0.77**	0.04	−0.02	0	0.08
(E1) Raise head	0.09	0.07	0	0.04	0.19	**0.73**
(E1) Toss head	0.22	0.04	−0.01	−0.03	0.18	**0.63**
(E1) Pull on reins	0.06	0.07	0.05	0	0.08	**0.79**
(E1) Brace neck	0.01	0.02	0.11	0.06	0.05	**0.76**
(E1) Excited canter	0.2	0.06	−0.12	0.13	0.03	**0.58**
(E7) Head up bridled	0.11	0.12	0.03	−0.01	**0.74**	0.18
(E7) Pull back bridled	0.09	0.18	0.07	0.06	**0.79**	0.16
(E7) Pull back unbridled	−0.01	0.1	0.1	0.13	**0.68**	0.12
(E9) Catch field	−0.03	0	0.05	**0.83**	−0.04	0.09
(E9) Move catch	0.1	0.03	0.08	**0.76**	0.21	0.07
(T17) Verbal correction	**0.61**	0.14	0.44	−0.1	−0.04	0.09
(T17) Correct ridden	**0.68**	0.11	0.22	−0.04	−0.08	0.19
(T21) Round pen lunge	**0.66**	0.06	0.14	0.02	0.1	0.04
(T21) Canter lunge	**0.74**	0.04	−0.01	0.07	0.05	0.17
(T5) Hosed	**0.47**	0.12	−0.02	−0.08	0.32	0.01
(T15) Girthed	**0.46**	−0.01	0.18	0.14	0.03	0.13

**Table 4:** The two sets of results of the univariate analyses: the first is based on the sex of the survey respondent and the second is based on the frequency of handling by male humans. The values appearing in the three columns labelled sex of survey respondent were evaluated according to the sex of the respondent, while those on the right were evaluated according to the frequency with which the focal horse was handled by male humans. Non-index predictors with *p*-value < 0.3 appear in bold below. Bolded items were passed into the multivariate model building process.

Sex of the Survey Respondent				Frequency of Handling by Male Humans		
E-BARQ Item	Lr χ^2^	df	*p*-Value	Lr χ^2^	df	*p*-Value
Rider’s country	121.88	10	**<0.001**	30.979	10	**<0.001**
Rider’s age	40.399	7	**<0.001**	30.979	7	**0.080**
Rider’s laterality	6.4427	2	**0.040**	3.000	2	**0.223**
Rider experience	24.066	7	**0.001**	10.601	7	**0.157**
Sex of horse	26.034	4	**<0.001**	5.822	5	0.324
Age of horse	0.701	1	0.4026	2.762	1	**0.097**
Colour	26.978	10	**0.003**	10.120	10	0.430
Horse height	13.418	8	**0.098**	3.829	8	0.872
Breed	56.369	13	**<0.001**	53.703	13	**<0.001**
Discipline	59.369	19	**<0.001**	33.489	19	**0.021**
Human social confidence	0.195	1	0.659	0.621	1	0.431
Intervention compliance	1.318	1	**0.251**	0.177	1	0.674
Head compliance	7.2663	1	**0.007**	2.936	1	**0.087**
Bridling compliance	0.32745	1	0.567	0.073	1	0.786
Catch compliance	11.355	1	**<0.001**	0.652	1	0.42
Absence of defensive aggression	2.051	1	**0.152**	0.022	1	0.883

**Table 5:** Three traits were significantly different when male humans reported on their horses’ behaviour. Male humans were significantly more likely to report that horses were difficult to catch (*p*-value = 0.002) and more defensive when approached (*p*-value = 0.035), but less likely to report that horses pulled on the reins/braced the neck or tossed their head (*p*-value = 0.048).

Trait	Coefficient	Std. Error	*t*-Value	*p*-Value
Catching Compliance	−1.42	0.46	−3.11	0.002
Human Social Confidence	−0.839	0.399	−2.104	0.035
Head Compliance	0.667	0.337	1.980	0.048

## Data Availability

Not applicable.

## Supplementary Materials

The following are available online at https://www.mdpi.com/2076-2615/11/1/130/s1 Supplementary File S1—E-BARQ Survey.
