# Supplementary material for: The Impact of the Sex of Handlers and Riders on the Reported Social Confidence, Compliance and Touch Sensitivity of Horses in Their Care
Source: Animals (Basel). 2021 Jan 8;11(1):130. doi: 10.3390/ani11010130 (PMC7827593; doi:10.3390/ani11010130)
Supplement: Supplementary file 1 [file animals-11-00130-s001.pdf]

Data Handling and analysis for Ashley Anzulewicz

Project: <u>Confidence, sensitivity and compliance reported among horses with male vs. female riders</u>

Main Dependent variable(s) of interest:

- Gender of Survey respondent
- Extent of handling by men and boys

Main Predictor variables of interest:

Items loading strongly in preliminary EBARQ analysis onto

- E1
- E9
- P3
- P5
- P15
- P21
- P17
- P24

Independent Variables

<u>Determination of number and composition of Independent Indices</u>

<u>Parameterisation</u>

P3 Handing compliance e.g. :Stand for vet

Always = Score of 5→ 4→3→2→ Never= score of 1

P5: Defensive or aggressive when sponged or hosed; P15: Defensive or aggressive when girthed or saddled; P24 e.g.: Defensive or aggressive when approached by you in the paddock

No signs= score of 5 → 4→3→2→ Serious signs = score of 1

E1 e.g.: Raise head when ridden/driven; E7 e.g.: Head up when bridled; E9 Move away when being caught (but NOT catch field)

Never=5 → 4→3→2→ Always =1

E9 Easy to catch in field

Never=1 → 2→3→4→ Always =5

24 EBARQ items (once ridden and unridden questions are combined) were in the 8 underlying factors of interest.

To combine these into a smaller number of relatively uncorrelated indicies, a Parallel Analysis, comparing the scree of factors of the standardised observed data with that of a random data matrix of the same size, was used to assess the underlying number of factors in the 24 items using the psych package of R statistical software.

This Parallel Analysis suggested 6 underlying components. When six principal components were extracted using the psych package and rotated using a varimax rotation, to facilitate interpretation, the loadings were as follows.

	RC2	RC6	RC1	RC3	RC5	RC4
P24_approach_paddock	0.08	-0.04	0.16	0.77	0.1	0.2
P24_approach_stable	0.09	-0.01	0.12	0.84	0.06	0.01
P24_approach_you_eating	0.01	0.06	0.24	0.71	0.07	-0.02
P3_vet	0.74	0.1	0.07	0.07	0.18	-0.02
P3_dentist	0.66	0.06	0.08	-0.03	0.11	-0.03
P3_feet_picked	0.79	0	0.09	0.11	0.11	0.07
P3_feet_trimming	0.85	0	0.08	0.06	0.05	0.05
P3_shod	0.77	0.08	0.02	0.04	0	-0.02
E1_raise_head	0.07	0.73	0.09	0	0.19	0.04
E1_toss_head	0.04	0.63	0.22	-0.01	0.18	-0.03
E1_pull_on_reins	0.07	0.79	0.06	0.05	0.08	0
E1_brace_neck	0.02	0.76	0.01	0.11	0.05	0.06
E1_excited_canter	0.06	0.58	0.2	-0.12	0.03	0.13
E7_head_up_bridled	0.12	0.18	0.11	0.03	0.74	-0.01
E7_pull_back_bridled	0.18	0.16	0.09	0.07	0.79	0.06
E7_pull_back_unbridled	0.1	0.12	-0.01	0.1	0.68	0.13
E9_catch_field	0	0.09	-0.03	0.05	-0.04	0.83
E9_move_catch	0.03	0.07	0.1	0.08	0.21	0.76
P17_verbal_correction	0.14	0.09	0.61	0.44	-0.04	-0.1
P_17correct_ridden	0.11	0.19	0.68	0.22	-0.08	-0.04
P21_round_pen_lunge	0.06	0.04	0.66	0.14	0.1	0.02
P_21canter_lunge	0.04	0.17	0.74	-0.01	0.05	0.07
P5_hosed	0.12	0.01	0.47	-0.02	0.32	-0.08
P15_girthed	-0.01	0.13	0.46	0.18	0.03	0.14

Kaiser, Meyer, Olkin Measures of Sampling Adequacy (MSA) and Cronbach’s alpha (α) were also considered using the psych package.

The overall MSA values for P24 was 0.66 and Cronbach’s alpha was 0.75 (95% Confidence interval= 0.73 - 0.77), the latter of which was not improved by dropping any item. The overall MSA value for P3 was 0.75 and Cronbach’s alpha was 0.83 (95% Confidence interval= 0.82 - 0.84), the latter of

which was not improved by dropping any item. The overall MSA value for E1 was 0.78 and Cronbach's alpha was 0.77 (95% Confidence interval= 0.75 - 0.78), the latter of which was not improved by dropping any item. The overall MSA value for E7 was 0.63 and Cronbach's alpha was 0.77 (95% Confidence interval= 0.68 - 0.73). Removing E7_pull_back _unbridled slightly lifted the alpha to 0.71, given the small improvement relative to the confidence interval, and the small number of items in the index, the item was retained. The overall MSA value for 91 was 0.50 and Cronbach's alpha was 0.54 (95% Confidence interval= 0.5 – 0.58), the latter of which was not improved by dropping any item. Lastly, the defensive/aggression factors (P5, P15, P17, P21) had an overall MSA value of 0.71 and Cronbach's alpha was 0.72 (95% Confidence interval= 0.70 - 0.74), the latter of which was not improved by dropping any item.

Construction of Independent Indices

Indexes were constructed by assigning a numerical value to scores on the Likert scale (see Parameterisation above) of the relevant EBARQ items, and summing these values together. In the case of missing values, the sum was divided by the number of EBARQ items in the index for which information for that horse was available and multiplied by the number of items used to calculate the index, weighting the missing value according to the horse's score for similar items rather than imputing an overall mean. If no EBARQ items for an index were completed then a value for that horse was not calculated.

P24 → Human Social Confidence index

P3→ Intervention compliance index

E1→ Head compliance index

E7 → Bridling compliance index

E9→ Catch compliance index

P5, P15, P17, P21 -→ Defensive Aggression index

Boxplots of these indices are shown below.

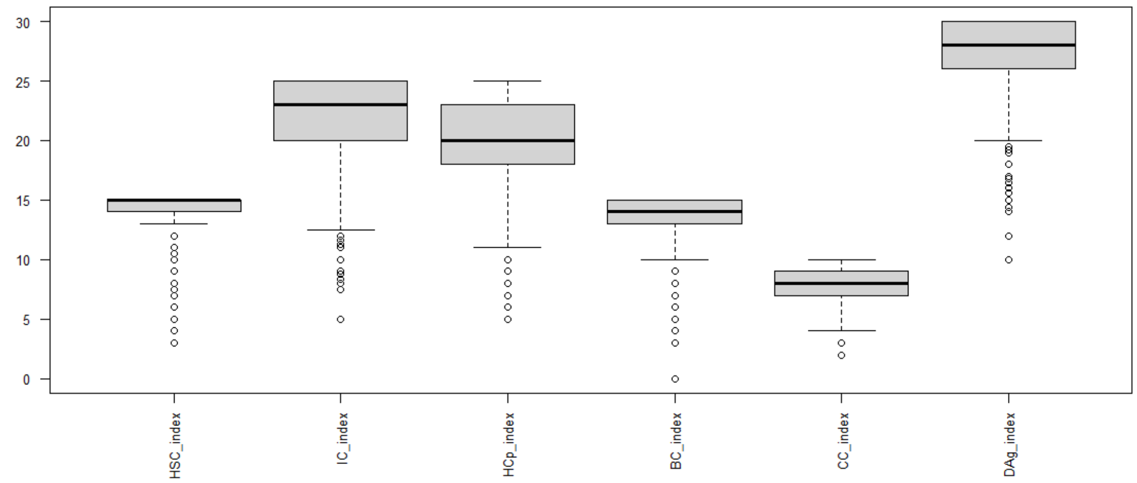

The data was inverted and log transformed to lessen skew (see below), and then uninverted for ease of interpretation of coefficients.

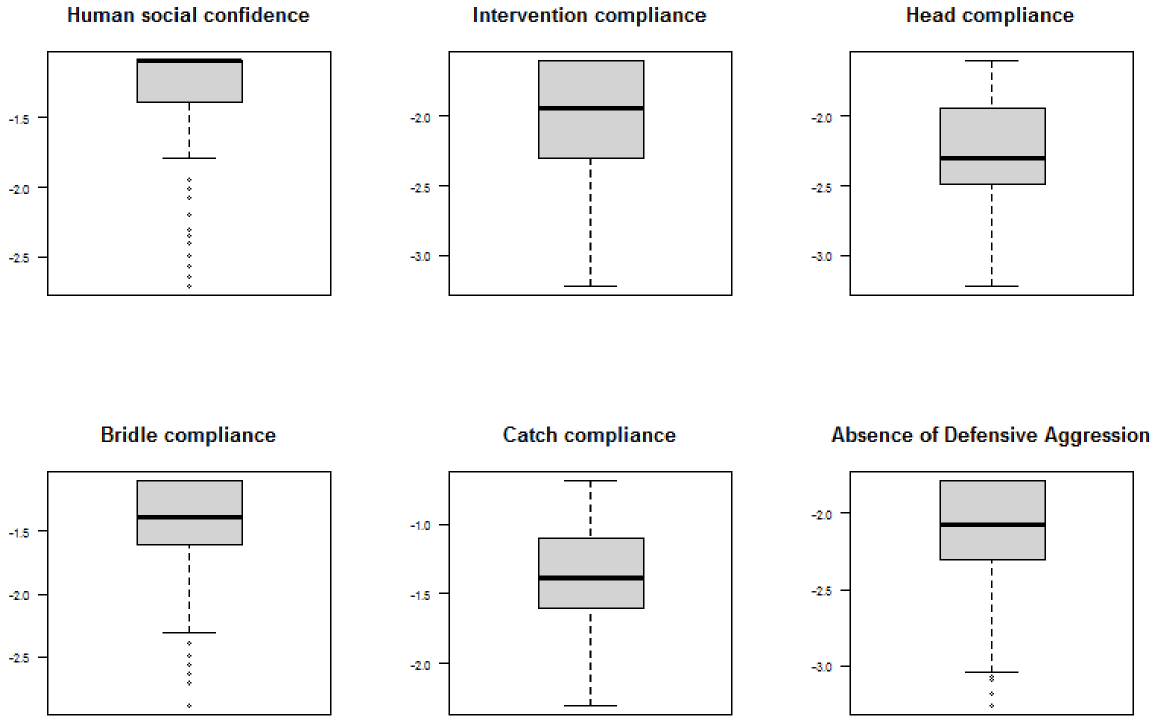

Other Independent / Predictor/ Explanatory Variables

Other predictor variables available (including potential confounders) were assessed for potential inclusion in the final model by univariate analysis, along with the above indexes See below.

	Gender of survey taker			Frequency Men Boys Handling		
	LR χ^2	df	P value	LR χ^2	df	P value
Country of Rider	121.88	10	<0.001	30.979	10	<0.001
Age of Rider	40.399	7	<0.001	30.979	7	0.080
laterality of Rider	6.4427	2	0.040	3.000	2	0.223
Sex of Horse	26.034	4	<0.001	5.822	5	0.324
Age of horse	0.701	1	0.4026	2.762	1	0.097
Colour	26.978	10	0.003	10.120	10	0.430
Height_Horse	13.418	8	0.098	3.829	8	0.872
Breed	56.369	13	<0.001	53.703	13	<0.001
Rider Experience	24.066	7	0.001	10.601	7	0.157
Discipline	59.451	19	<0.001	33.489	19	0.021
Human Social Confidence	0.195	1	0.659	0.621	1	0.431
Intervention Compliance	1.318	1	0.251	0.177	1	0.674
Head Compliance	7.2663	1	0.007	2.936	1	0.087
Bridling Compliance	0.32745	1	0.567	0.073	1	0.786
Catch Compliance	11.355	1	<0.001	0.652	1	0.42
Absence of Defensive Aggression	2.051	1	0.152	0.022	1	0.883

Non-index predictors with P< 0.3 on univariate analysis were passed into the multivariate model building process, apart from Breed, which, because of strong multi-collinearity with Discipline was discarded.

Index predictors with P< 0.7 were passed into the multivariate model building process,

Multivariable Model Building

Gender of Respondent Model

Dependent Variable: Gender of Survey Taker

Potential Explanatory Variables: Human Social Confidence index, Intervention Compliance Index, Head compliance Index, Bridling Compliance Index, Catch Compliance Index, Absence of Defensive Aggression Index.

Country of Rider, Age of Rider, laterality of Rider, Sex of Horse, Colour, Height_Horse, Rider Experience, Discipline

Stepwise model reduction (removing least significant term)

Full model AIC: 452.0184

Remove Absence of Defensive Agression Index AIC: 450.0887

Remove Height Horse AIC: 446.5283

Remove Human Social Confidence Index AIC: 444.9575

Remove Sex of Horse AIC: **442.1068**

Remove Laterality AIC: 447.2531

Restore Laterality

Trial Remove Intervention Compliance index AIC: 442.1854

Restore Intervention Compliance index

Final model is a logistic regression using the stats package

```
Final Model: Final_Model<- glm(Gender ~ Analysis_country+
        Age+
        laterality+
        Analysis_colour+
        Analysis_Discipline+
        Experience+
        BC+
        IC+
        HCp+
        CC, data = mydata, family = "binomial")
```

Residuals were assessed graphically.

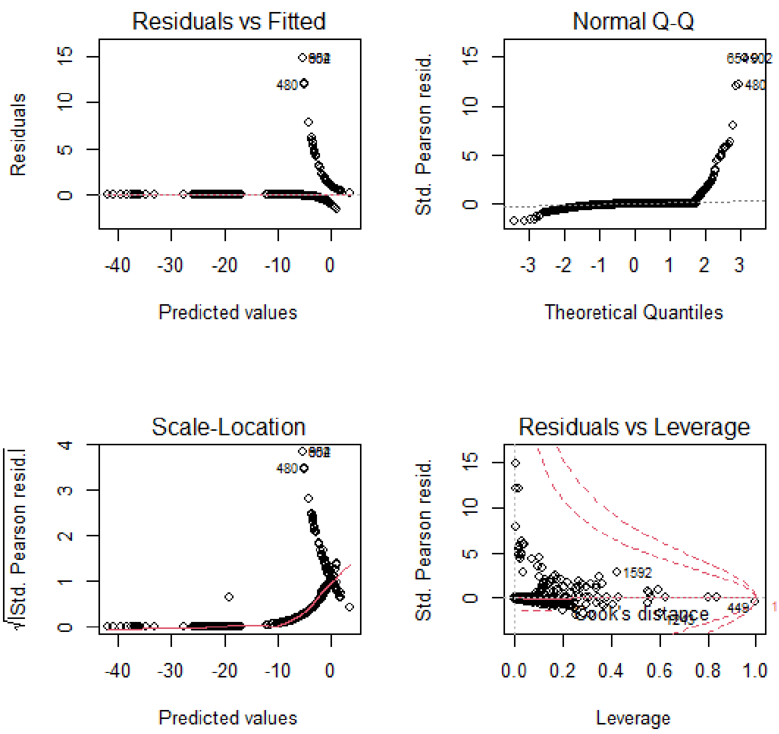

Coefficients

(Remember that these are on the logit scale!)

(Intercept)	-4.84	1.64	-2.96	0.003
Belgium (ref Australia)	2.42	0.91	2.66	0.008
Canada	-0.18	0.71	-0.26	0.796
Italy	3.22	0.90	3.57	0.000
Mexico	2.91	0.82	3.55	0.000
NewZealand	-16.61	1231.00	-0.01	0.989
Other	3.03	0.48	6.27	0.000
SouthAfrica	-18.27	2850.00	-0.01	0.995
Sweden	-17.61	3446.00	-0.01	0.996
UK	-1.55	1.37	-1.13	0.258
USA	1.17	0.49	2.39	0.017
Age25-34 (ref 18-24)	-0.42	0.89	-0.48	0.633
Age35-44	0.52	0.67	0.78	0.438
Age45-54	0.89	0.62	1.43	0.154
Age55-64	2.01	0.63	3.18	0.001
Age65-74	1.97	0.74	2.65	0.008
Age75	6.94	1.61	4.31	0.000
AgeUnder18	-0.20	1.32	-0.16	0.877
lateralityI'm	-15.59	3149.00	-0.01	0.996
lateralityLEFT	0.66	0.51	1.29	0.199

Analysis_colourBlack	-0.26	0.65	-0.40	0.687
Analysis_colourBrown	1.37	0.52	2.65	0.008
Analysis_colourChesnut	0.18	0.47	0.38	0.705
Analysis_colourDilution	-1.67	1.12	-1.49	0.136
Analysis_colourGrey	-1.00	0.69	-1.45	0.148
Analysis_colourLeopard	2.43	0.97	2.51	0.012
Analysis_colourPalamino	0.54	0.81	0.67	0.504
Analysis_colourRoaned	1.37	1.19	1.15	0.249
Analysis_colourWhite	1.56	2.25	0.70	0.486
Analysis_colourWhitepatterned	-0.67	0.85	-0.79	0.432
Adult riding Club (ref= Pleasure horse)	0.12	1.13	0.11	0.913
Breeding_conformation	2.10	0.85	2.47	0.014
Companion Horse	-2.87	1.68	-1.71	0.087
Competitive_riding	1.55	0.68	2.29	0.022
Dressage	0.59	0.51	1.14	0.254
Endurance	2.56	1.00	2.56	0.011
Equitation	-18.09	3057.00	-0.01	0.995
Eventing	-0.93	1.12	-0.83	0.407
Liberty	-0.06	1.24	-0.05	0.964
Mounted_games	-16.95	3133.00	-0.01	0.996
Other	-2.38	2.23	-1.07	0.286
Pony Club	-16.24	1993.00	-0.01	0.993
Racing	2.65	1.08	2.47	0.014
Show Jumping	-0.30	0.79	-0.38	0.705
Therapy_horse	-16.57	4610.00	0.00	0.997
Trail Riding/Hacking	0.36	0.58	0.62	0.532
Western_events	-0.33	0.93	-0.36	0.720
Working Equitation	-17.19	3444.00	-0.01	0.996
Working_horse	0.05	1.31	0.04	0.969
Experience_Most of life (ref= all of life)	0.47	0.46	1.04	0.300
No Experience	-13.38	8341.00	0.00	0.999
Experience >8 years	0.01	0.55	0.01	0.990
Experience <2 years	1.79	0.87	2.06	0.039
Experience <5 years	1.00	0.73	1.37	0.170
Experience <8 years	0.16	0.91	0.18	0.859
Experience <1 years	4.12	0.82	5.05	0.000
IC	0.50	0.46	1.10	0.273
BC	0.52	0.52	1.01	0.315
HCp	0.64	0.52	1.21	0.225
CC	-1.42	0.46	-3.11	0.002

Model Fit statistics

Null deviance: 563.75 on 1688 degrees of freedom
Residual deviance: 322.11 on 1629 degrees of freedom
(313 observations deleted due to missingness)
AIC: 442.11

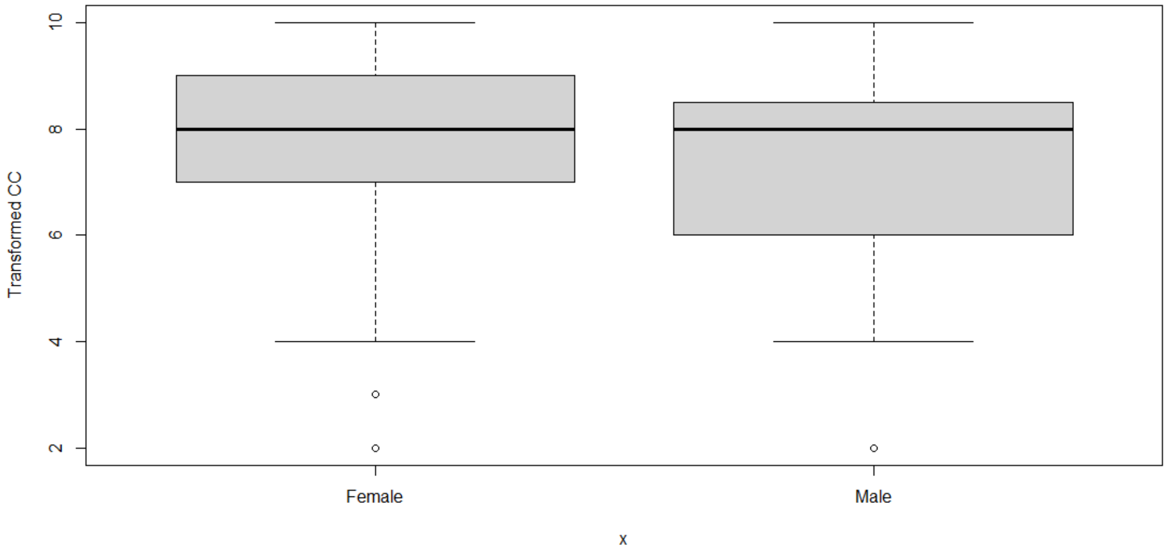

Frequency of Male Handling Model

Dependent Variable: Gender of Survey Taker

Potential Explanatory Variables: Human Social Confidence index, Intervention Compliance Index, Head compliance Index, Bridling Compliance Index, Catch Compliance Index, Absence of Defensive Aggression Index.

Country of Rider, Age of Rider, laterality of Rider, Age of horse, Rider Experience, Discipline

Frequency of handling by women/girls will also be added to control for frequency of handling itself.

Full model AIC: 858.8594

Drop Intervention Compliance: 856.8598

Drop Bridle Compliance: 855.0370

Drop Experience: 847.8838

Drop Laterality: 847.5389

Drop Horse age: 848.7478

Restore Horse age

Final Model: An ordinal logistic regression using the MASS package

```
Final_model2 <- polr(Handle_male2 ~ Analysis_country+
                   Age+
                   Analysis_Discipline+
                   HSC+
                   HCp+
                   Handle_female2,
                   data = mydata,  Hess=TRUE)
```

The parallel log odds assumption was assessed graphically and using a likelihood ratio test using the ordinal package. Surrogate residuals were generated by the sure package and assessed graphically.

Coefficients

(Remember that these are on the logit scale!)

	Coefficient	SE	T	P
Analysis_country2Canada	0.623	0.438	1.423	0.155
Analysis_country2Italy	-1.534	1.258	-1.219	0.223
Analysis_country2Mexico	1.395	1.290	1.082	0.279
Analysis_country2New_Zealand	-1.564	0.646	-2.421	0.015
Analysis_country2Other	0.868	0.478	1.816	0.069
Analysis_country2South_Africa	-0.322	1.086	-0.297	0.766
Analysis_country2UK	-0.075	0.419	-0.178	0.858
Analysis_country2USA	0.732	0.379	1.931	0.053
Age<18	1.989	1.227	1.621	0.105
Age25-34	0.453	0.466	0.972	0.331
Age35-44	-0.104	0.475	-0.219	0.826
Age45-54	-0.142	0.425	-0.334	0.738
Age55-64	0.561	0.445	1.260	0.208
Age65-74	1.489	0.580	2.565	0.010
Age75	1.635	1.286	1.271	0.204
Age_horse	-0.035	0.018	-1.985	0.047
Analysis_DisciplineAdult_riding_club	-0.156	0.708	-0.220	0.826
Analysis_DisciplineBreeding_conformation	1.611	0.515	3.130	0.002
Analysis_DisciplineCompanion_horse\t	0.115	0.453	0.254	0.800
Analysis_DisciplineCompetitive_riding	1.044	0.776	1.347	0.178
Analysis_DisciplineDressage	0.117	0.507	0.230	0.818
Analysis_DisciplineEndurance	-0.618	0.975	-0.634	0.526
Analysis_DisciplineEquitation	-0.328	2.046	-0.160	0.873
Analysis_DisciplineEventing	0.212	0.902	0.235	0.814
Analysis_DisciplineLiberty	0.582	0.637	0.914	0.360
Analysis_DisciplineMounted_games	-1.135	1.508	-0.753	0.452
Analysis_DisciplineOther	-0.518	0.426	-1.217	0.224
Analysis_DisciplinePony_club	-0.044	1.359	-0.032	0.974
Analysis_DisciplineRacing	0.910	1.266	0.719	0.472
Analysis_DisciplineShow-jumping	0.817	0.681	1.200	0.230
Analysis_DisciplineTherapy_horse	-1.148	0.978	-1.174	0.240
Analysis_DisciplineTrail_riding_hacking	-0.116	0.529	-0.220	0.826
Analysis_DisciplineWestern_events	-0.594	0.710	-0.837	0.403
Analysis_DisciplineWorking	-0.043	1.107	-0.038	0.969
Analysis_DisciplineWorking_horse	-0.586	1.029	-0.570	0.569

HSC	-0.839	0.399	-2.104	0.035
HCp	0.667	0.337	1.980	0.048
Handle_female2B	-0.358	0.708	-0.505	0.614
Handle_female2C	-0.347	0.877	-0.396	0.692
Handle_female2D	-0.634	0.765	-0.829	0.407
Handle_female2E	0.037	0.719	0.052	0.959
Handle_female2F	0.515	0.677	0.761	0.447
A|B	-0.778	1.194	-0.652	0.515
B|C	0.551	1.196	0.461	0.645
C|D	0.748	1.197	0.625	0.532
D|E	1.378	1.199	1.149	0.250
E|F	2.162	1.201	1.800	0.072

Some graphs (remember x axis are transformed)

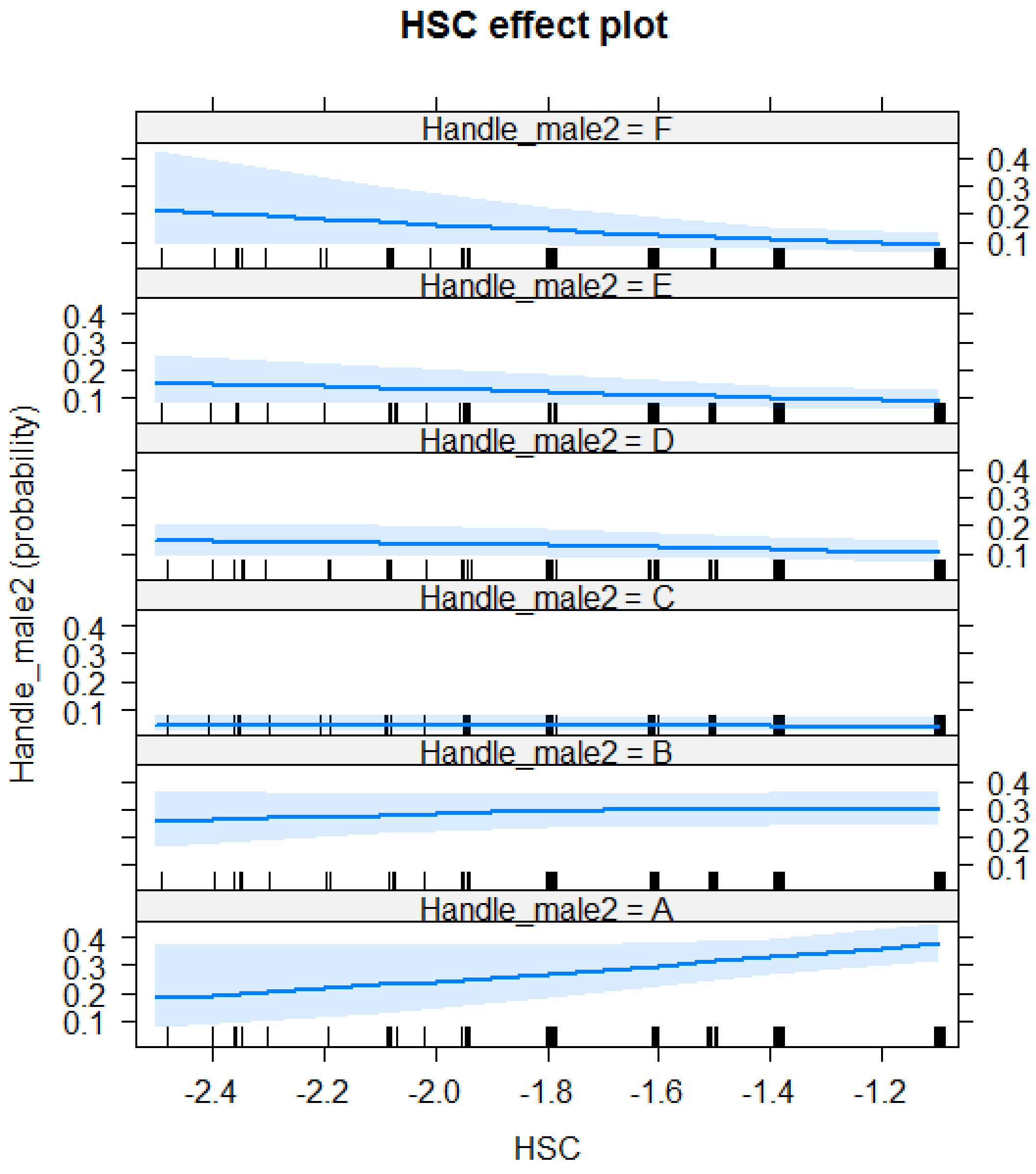

Where A =Never, B = 1-6 times in 6 months, C=Monthly, D= Fortnightly to weekly, E= Several times a week, F= Daily

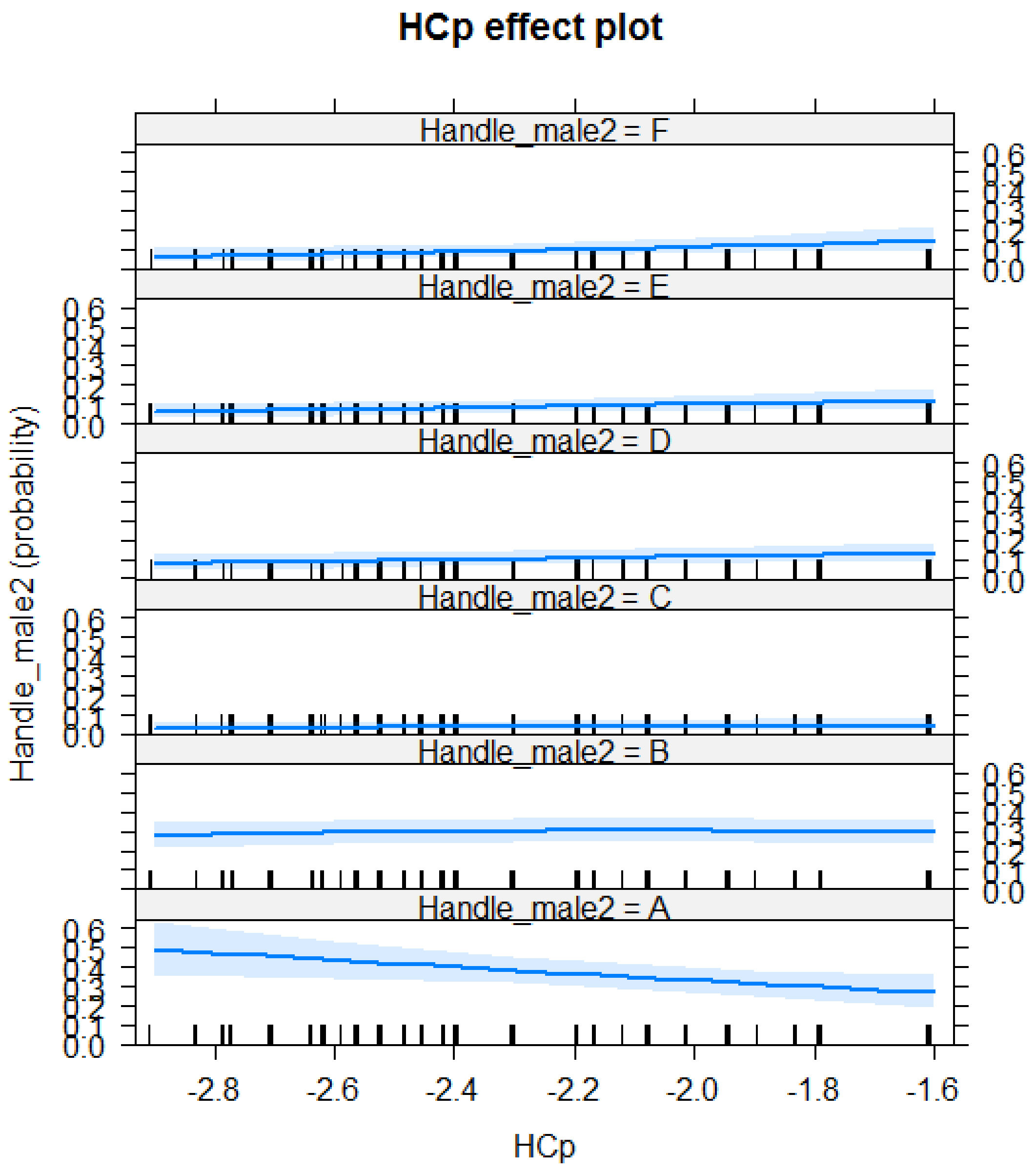

Where A =Never, B = 1-6 times in 6 months, C=Monthly, D= Fortnightly to weekly, E= Several times a week, F= Daily
